# Omicron variants escape the persistent SARS-CoV-2-specific antibody response in 2-year COVID-19 convalescents regardless of vaccination

**DOI:** 10.1080/22221751.2022.2151381

**Published:** 2022-12-28

**Authors:** Miao Wang, Bing Zhou, Qing Fan, Xinrong Zhou, Xuejiao Liao, Jingyan Lin, Zhenghua Ma, Jingke Dong, Haiyan Wang, Xiangyang Ge, Bin Ju, Zheng Zhang

**Affiliations:** a Institute for Hepatology, National Clinical Research Center for Infectious Disease, Shenzhen Third People’s Hospital, The Second Affiliated Hospital, School of Medicine, Southern University of Science and Technology, Shenzhen, People’s Republic of China; b Guangdong Key Laboratory for Anti-infection Drug Quality Evaluation, Shenzhen, People’s Republic of China; c Shenzhen Research Center for Communicable Disease Diagnosis and Treatment of Chinese Academy of Medical Science, Shenzhen, People’s Republic of China

**Keywords:** SARS-CoV-2, Omicron variants, COVID-19 convalescent, 2 years after discharge, antibody response, inactivated vaccines

## Abstract

With the ongoing COVID-19 pandemic and the emergence of various SARS-CoV-2 variants, a comprehensive evaluation of long-term efficacy of antibody response in convalescent individuals is urgently needed. Several longitudinal studies had reported the antibody dynamics after SARS-CoV-2 acute infection, but the follow-up was mostly limited to 1 year or 18 months at the maximum. In this study, we investigated the durability, potency, and susceptibility to immune evasion of SARS-CoV-2-specific antibody in COVID-19 convalescents for 2 years after discharge. These results showed the persistent antibody-dependent immunity could protect against the WT and Delta variant to some extent. However, the Omicron variants (BA.1, BA.2, and BA.4/5) largely escaped this preexisting immunity in recovered individuals. Furthermore, we revealed that inactivated vaccines (BBIBP-CorV, CoronaVac, or KCONVAC) could improve the plasma neutralization and help to maintain the broadly neutralizing antibodies at a certain level. Notably, with the time-dependent decline of antibody, 1-dose or 2-dose vaccination strategy seemed not to be enough to provide immune protection against the emerging variants. Overall, these results facilitated our understanding of SARS-CoV-2-induced antibody memory, contributing to the development of immunization strategy against SARS-CoV-2 variants for such a large number of COVID-19 survivors.

## Introduction

Coronavirus disease 2019 (COVID-19), caused by severe acute respiratory syndrome coronavirus 2 (SARS-CoV-2), has been a global pandemic lasting for more than two years. As of October 2022, the number of people infected with SARS-CoV-2 has exceeded 626 million globally, and approximately 6.57 million cumulative deaths have been reported [1]. Many studies have shown that the antibody-mediated protective immunity induced either by the natural infection or vaccination is of great importance for the control of the COVID-19 pandemic. After natural infection, most COVID-19 patients could produce virus-specific IgM, IgA, and IgG antibodies within a few days [2–6]. Then, the level of IgG antibodies kept increasing for 1 month after symptom onset, declined after 2 months, and even remained detectable over 18 months [7–10]. Recently, a longitudinal cohort study reported on the evolution of health outcomes among the survivors after 2 years of SARS-CoV-2 infection in Wuhan, and found that they had significantly lower health status than the general population [11]. However, SARS-CoV-2-specific antibody response and neutralizing activity are largely unknown in COVID-19 convalescents, who have been discharged from hospital for 2 years. Currently, there is still no follow-up study with such long duration. Therefore, this kind of long-term study on the duration of functional neutralizing antibody response after SARS-CoV-2 infection is very necessary, which is of great significance to pandemic control and prevention as well as vaccine development.

In addition, multiple SARS-CoV-2 vaccines have been approved globally, and vaccination is effective in reducing the morbidity and mortality among infected individuals [12–15]. Especially, in order to fight against breakthrough infection caused by new variants, the number of vaccine doses have been increased to as many as four. This kind of 4-dose vaccination strategy has been reported to provide protection against SARS-CoV-2 infection in several studies [16–19]. Therefore, several countries, such as the United States, Israel, and the United Kingdom [20], have begun to initiate the fourth dose of vaccination for high-risk people. Previously, we demonstrated that immunization with inactivated vaccine could significantly improve the broad neutralization of plasma against various SARS-CoV-2 variants in COVID-19 convalescent individuals [21], with some questions remaining about the duration of protective immunity and the necessity of the second dose or even the third dose of booster shot.

In this study, we aimed to assess the presence of SARS-CoV-2-specific antibodies, particularly broadly neutralizing antibodies, in individuals who have recovered from COVID-19 for up to 2 years, and to reveal the effects of inactivated vaccination on the plasma antibody level and neutralizing activities against the current SARS-CoV-2 variants. Our finding is critical for evaluating the potency and persistence of SARS-CoV-2 natural infection- and/or vaccine-induced protective antibody, will helping to guide updated vaccination strategy to prevent reinfection caused by SARS-CoV-2 variants in convalescents.

## Materials and methods

### Ethics

This study was approved by the Ethics Committee of Shenzhen Third People’s Hospital (approval number: 2021-030), and each participant signed an informed statement for sample collection and subsequent analysis.

### Study participants and sample collection

A longitudinal observational study was conducted on patients admitted to Shenzhen Third People’s Hospital from January to April 2020 who were diagnosed with SARS-CoV-2 infection by PCR test, and aimed to analyze the presence of specific antibodies against SARS-CoV-2. Based on the clinical characteristics during hospitalization, patients were classified into moderate, mild, severe, and critically ill, noted as group 1, 2, 3, and 4, respectively. In the following analyses, they were redistributed into two categories, including mild patients (group 1–2) and severe patients (group 3–4). During hospitalization and follow-up, all plasma and peripheral blood mononuclear cells (PBMCs) samples of enrolled patients were collected and stored at the BioBank of the Shenzhen Third People’s Hospital. Two years after discharge, 84 individuals were recruited for follow-up and blood donation. Plasma samples were heat-inactivated at 56°C for 1 h before use. The SARS-CoV-2 receptor binding domain (RBD)-specific plasma IgG, IgM, and IgA were measured using the Chemiluminescence Immunoassay Kits (Beijing Wantai) [22], displayed in the cut-off-index (COI). According to the manufacturer’s instructions, the positive thresholds of anti-RBD IgG, IgM, and IgA were 1 [7].

### Flow cytometric analysis of RBD-specific memory B cells

Firstly, thawed PBMCs were stained with the antibody cocktail consisting of CD19: PE-Cy7, CD3: Pacific Blue, CD8: Pacific Blue, CD14: Pacific Blue, CD27: APC-H7, and IgG: FITC (BD Biosciences) to gate IgG + memory B cells. To target the antigen-specific B cells, SARS-CoV-2 wild-type RBD with His tag (Sino Biological) was also added and used as a probe. Then, two anti-His secondary antibodies separately labelled with APC and PE (Abcam) were both used to recognize the RBD bait and exclude nonspecific staining. Dead cells were excluded using the LIVE/DEAD Fixable Dead Cell Stain Kit (Invitrogen). Flow cytometric data were acquired on an FACSymphony S6 flow cytometer (BD Biosciences) and analyzed using FlowJo software (TreeStar).

### SARS-CoV-2 pseudovirus-based neutralization assay

SARS-CoV-2 wild-type and variant pseudoviruses were generated by the co-transfection of HEK-293 T cells with corresponding spike-expressing plasmid and the env-deficient HIV-1 backbone vector (pNL4-3.Luc.R-E-) [21]. After 2 days of incubation, the supernatant was collected, centrifuged, filtered to obtain pseudovirus, and stored at −80°C. For determining neutralizing activity, 3-fold serially diluted plasma samples (initial dilution 1:30) were incubated with an equal volume of diluted pseudovirus at 37°C for 1 h and then added to the HEK-293T-hACE2 cells. After 48 h incubation, the medium was removed and 100 μL of Bright-Lite Luciferase reagent (Vazyme Biotech) was added and allowed to stand at room temperature for 2 min, followed by measurement of luciferase activity using a Varioskan LUX multimode enzyme marker (Thermo Fisher Scientific, US). The 50% inhibitory dilution (ID_50_) was calculated using GraphPad Prism 8.0.2 software by log (inhibitor) vs. normalized response – Variable slope (four parameters) model. The limit of detection was 1:30. Any sample that does not neutralize SARS-CoV-2 at 30-fold dilution was given a value of 30 for representation and data analysis.

### Statistical analysis

All analyses were performed using R v4.0.3 software. To model how antibody response vary over time, a nonlinear regression analysis was performed with the ggplot2 package. The correlation between neutralizing titres and the days after the last vaccination was performed with a non-parametric Spearman’s correlation analysis. Levels of anti-RBD IgG and plasma neutralization were compared by two-tailed unpaired or paired Wilcoxon test and the two-tailed Kruskal–Wallis test with Dunn’s multiple-comparisons. *P < 0.05; **P < 0.01; ***P < 0.001; ****P < 0.0001.

## Results

### Characteristics of the cohort

**Figure 1.**
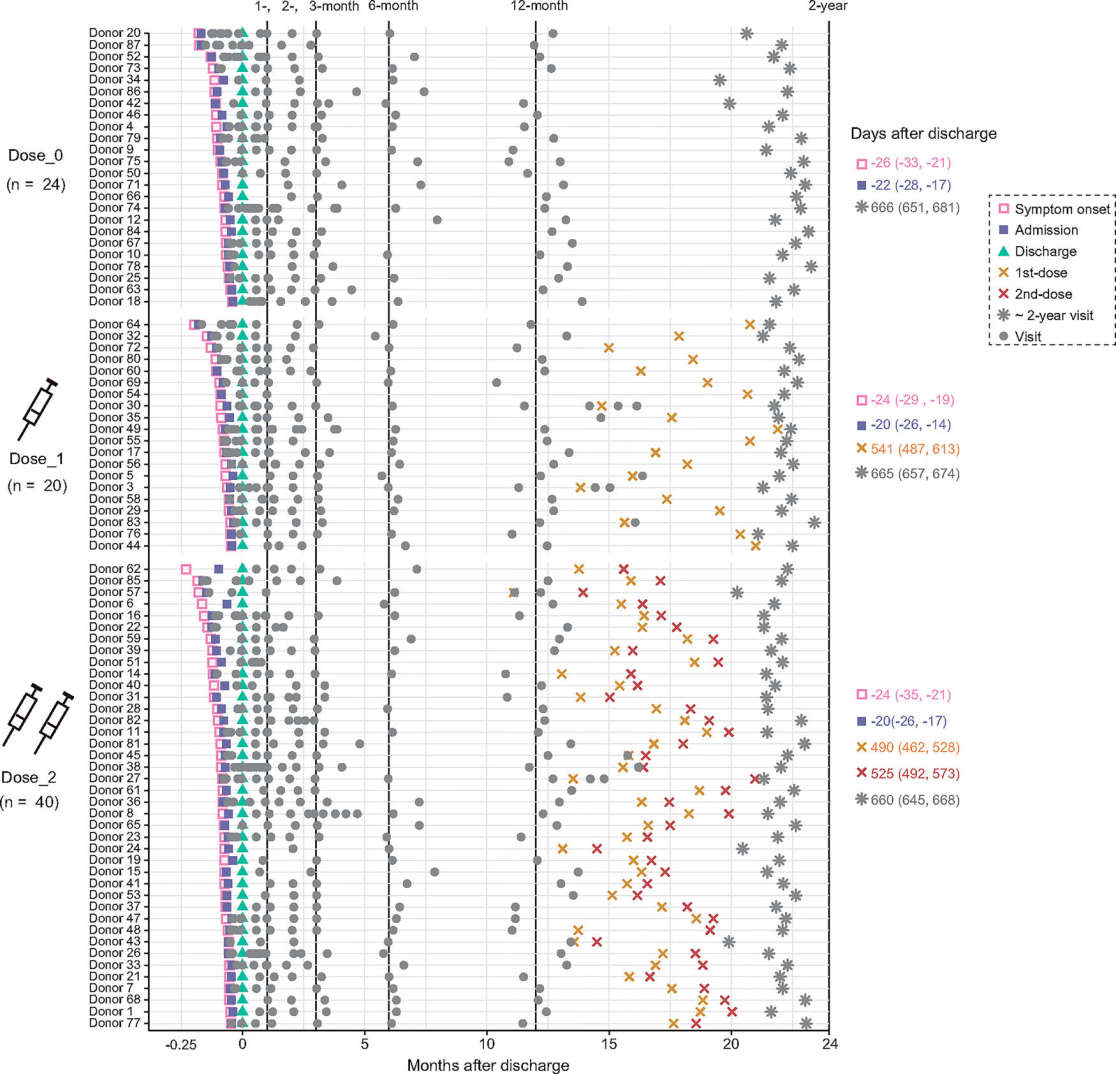
Timeline of events during hospitalization and follow-up. Key events included the symptom onset, admission, discharge, vaccination, and 2-year follow-up visit after discharge, with the discharge date normalized to Day 0 and the median time (IQR) to each stage marked on the right. Grey dots indicate samples collected at each time point.

Based on the vaccination status after discharge, 84 COVID-19 convalescents with two years of follow-up visit were divided into three groups, including 24 unvaccinated individuals (Dose_0), 20 individuals with 1-dose vaccination (Dose_1), and 40 individuals with 2-dose vaccination (Dose_2) until the last follow-up visit (Figure 1, Table S1). A total of 60 participants were immunized with SARS-CoV-2 inactivated vaccines, including BBIBP-CorV, CoronaVac, and KCONVAC. This cohort consisted of 55% (46/84) males, with a median age at discharge of 43 years old ranging from 1 to 69. A total of 748 plasma samples were collected from these 84 individuals during their hospitalization and at six follow-up visits after discharge (approximately 1-, 2-, 3-, 6-, 12-month, and 2-year). The median time at 2-year follow-up visit was 662 days (interquartile range (IQR): 647–674) after discharge. The median time interval from the last vaccination to the 2-year follow-up was 130 days (IQR: 45–177) and 136 days (IQR: 94-166) for the Dose_1 and Dose_2 groups, respectively.

### Dynamics of SARS-CoV-2 specific antibodies in convalescents throughout 2 years after discharge

**Figure 2.**
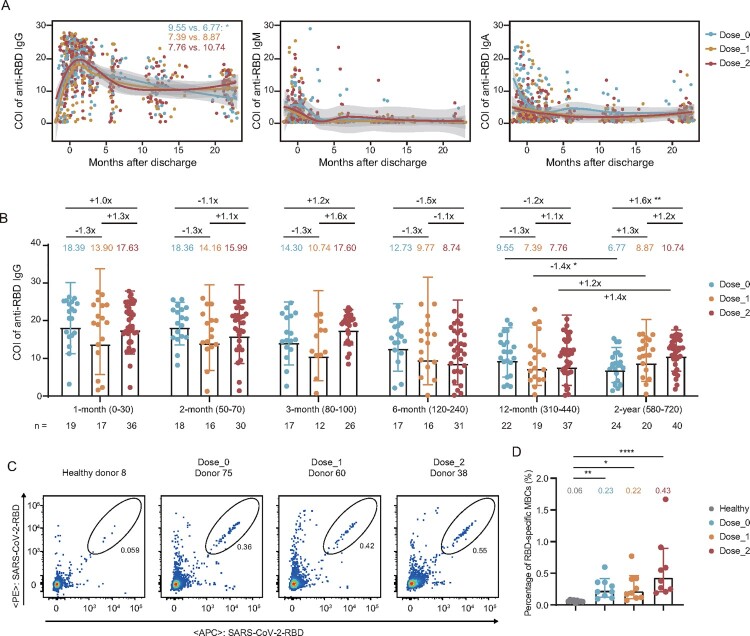
Longitudinal dynamics of plasma IgG, IgM, and IgA specifically binding to the WT SARS-CoV-2 RBD. (A) Kinetic of RBD-specific plasma IgG, IgM, and IgA from admission to 2 years post-discharge in 84 convalescent individuals grouped by the dose of inactivated vaccination. The level of plasma antibody is shown as a cut-off index (COI), and the value ≥1 is considered positive. (B) Comparison of the levels of anti-RBD IgG for the unvaccinated, 1-dose, and 2-dose groups at different follow-up time points. 1-month, 0–30 days after discharge (*n* = 72); 2-month, 50–70 days after discharge (*n* = 64); 3-month, 80–100 days after discharge (*n* = 55); 6-month, 120–240 days after discharge (*n *= 64); 12-month, 310–440 days after discharge (*n* = 78); 2-year, 580–720 days after discharge (*n* = 84). The number of samples for each group at each time point was labelled at the bottom. The geometric mean, fold-change, and significance of difference were labelled on the top. “−” represents decreased antibody level, and “+” represents increased antibody level. (C) The typical display of SARS-CoV-2-RBD-specific MBCs (CD19^+^CD3^−^CD8^−^CD14^−^CD27^+^IgG^+^RBD^+^) of one representative donor from each group. (D) The percentage of RBD-specific MBCs of 9 randomly selected donors in each of the non-vaccinated healthy donors, Dose_0, Dose_1, and Dose_2 groups. Geometric mean percentage and significance of difference were labelled on the top. Statistical analysis was performed using the two-tailed Kruskal-Wallis test with Dunn’s multiple-comparison test. ****P < 0.0001; **P < 0.01; *P < 0.05.

To characterize the kinetics of antibody responses across the whole disease process and up to 2 years after discharge, we tested the levels of IgG, IgM, and IgA binding to the SARS-CoV-2 RBD from the consecutive samples of 84 individuals in three groups (Figure 2(A)). These results showed that the specific IgG peaked at about 1 month after discharge and then began to constantly decline up to 12 months, because all participants were not immunized with any SARS-CoV-2 vaccines during this period. After that, a part of participants (60/84) received one or two doses of vaccines. Two years after discharge, the geometric mean value of IgG in unvaccinated individuals was further dropped to 6.77, which was significantly lower than that at the 12-month visit (9.55, *P *= 0.02). However, in the other two groups, immunization with one or two doses of inactivated vaccines prevented and even reversed the decline of anti-RBD IgG antibodies at 2-year visit (8.87 vs. 7.39 for Dose_1 group and 10.74 vs. 7.76 for Dose_2 group, respectively) (Figure 2(A), Figure S1). Three months after discharge, anti-RBD IgM became undetectable in most patients and the IgA were maintained at low levels for a long time. By contrast, despite the decrease found in anti-RBD IgG, it remained detectable and persisted from the 6-month to the 2-year visit after discharge, emphasizing the more important role of IgG in providing long-term protection against SARS-CoV-2 infection.

To better evaluate the contribution of vaccination to improvement in antibody value, we performed a quantitative analysis of anti-RBD IgG dynamics in the unvaccinated and vaccinated individuals and compared them at each time point of follow-up (Table S2). As shown in Figure 2(B), IgG values were similar in all three groups during 1 year after discharge (before vaccination). However, the values were significantly higher in patients who received two doses of SARS-CoV-2 inactivated vaccines, compared with that in the unvaccinated group at the 2-year visit. This result showed that vaccination with inactivated vaccines could effectively enhance the antibody level in convalescent COVID-19 individuals.

Moreover, we randomly selected 9 individuals from each group at the 2-year visit and 9 non-vaccinated healthy donors to measure the SARS-CoV-2 RBD-specific memory B cells (MBCs) (Figure S2). As shown in Figure 2(C,D), COVID-19 convalescents in three groups retained significantly detectable levels of RBD-specific MBCs at 2 years after discharge. Particularly, despite individuals in the unvaccinated group were not immunized with any additional vaccines, they still had some levels of RBD-specific MBCs, also indicating that humoral memory could persist for at least 2 years after SARS-CoV-2 infection.

### Neutralization by convalescent plasma against SARS-CoV-2 variants at 2 years after discharge

**Figure 3.**
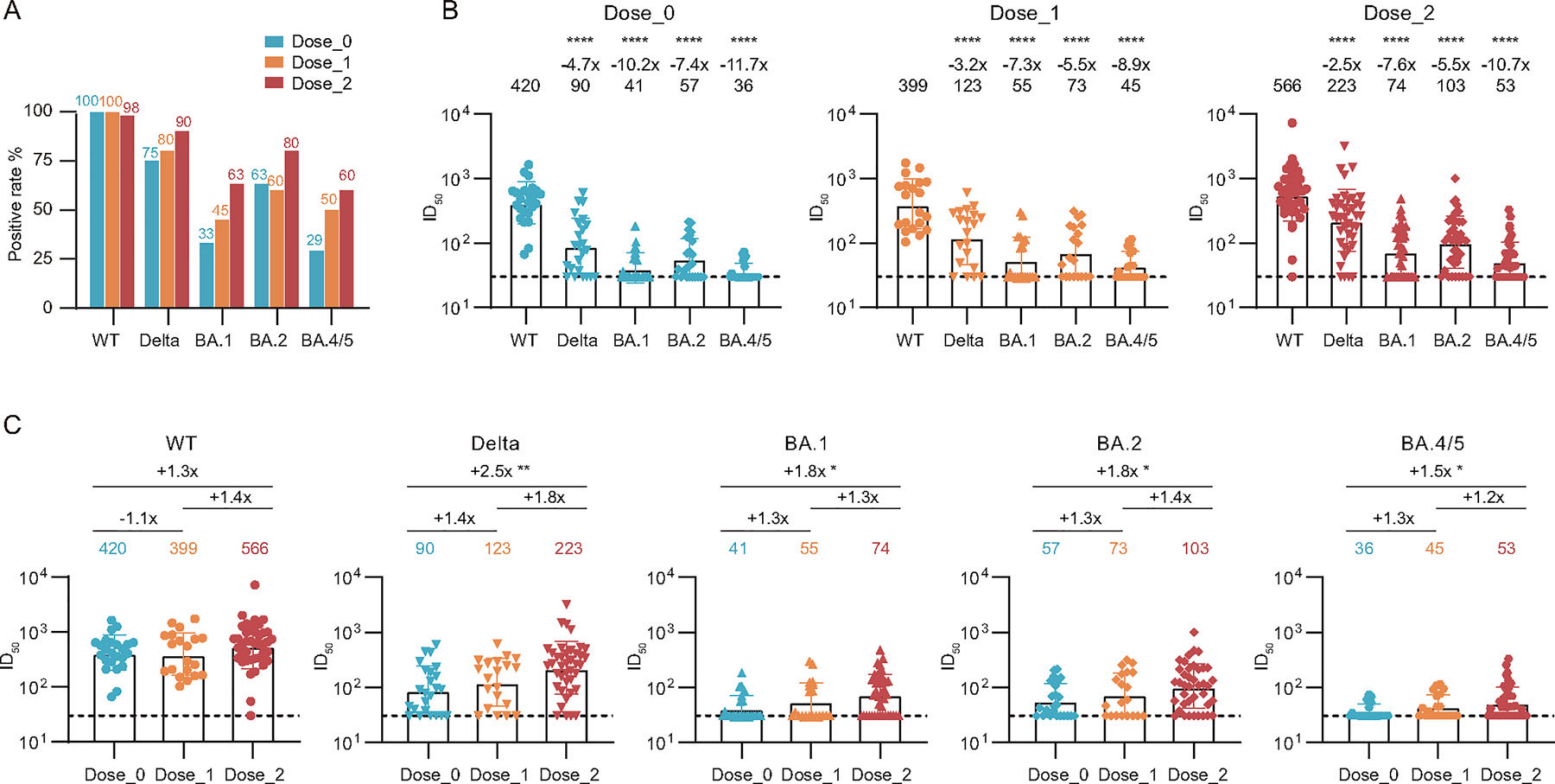
Effect of inactivated vaccination on the neutralizing antibody response among convalescents at the 2-year follow-up time point. (A) Percentage changes of positive plasma against WT SARS-CoV-2, Delta, and Omicron (BA.1, BA.2, and BA.4/5) variants. (B) The broad spectrum of plasma nAbs in unvaccinated (*n* = 24), 1-dose (*n *= 20), and 2-dose groups (*n *= 40). (C) Comparison of plasma neutralizing activities among different groups against WT SARS-CoV-2 and variants. The geometric mean, fold-change, and significance of difference were labelled on the top. “−” represents decreased neutralization activity, and “+” represents increased antibody level. Statistical analysis was performed using the two-tailed paired Wilcoxon test in (B) and the two-tailed Kruskal-Wallis test with Dunn’s multiple-comparison test in (C). ****P < 0.0001; **P < 0.01; *P < 0.05. The limit of detection was 1:30 dilution. ID_50_ indicates 50% inhibition dilution. The ID_50_ values are means of at least two independent experiments.

To evaluate the neutralization activity of the convalescent plasma at 2 years post discharge against the infection of major prevalent SARS-CoV-2 variants, we performed the pseudovirus-based neutralization assay to detect their abilities in inhibiting the virus entry, including wild-type (WT), Delta (B.1.617.2), and Omicron (BA.1, BA.2, and BA.4/5 (BA.4 and BA.5 sharing the same amino acid sequence in the spike)) variants (Figure 3, Figure S3, Table S3). On the whole, over 98% and 75% of plasma in three groups remained the neutralization against WT and Delta, respectively (Figure 3(A)). However, for three tested Omicron variants (BA.1, BA.2, and BA.4/5), the positive rate of neutralization was seriously decreased, especially in unvaccinated and 1-dose vaccination individuals. Compared with the neutralization against WT, all geometric mean titres (GMTs) of plasma neutralizing antibodies (nAbs) against tested variants were significantly decreased at the 2-year visit, with 420 for WT, 90 for Delta, 41 for BA.1, 57 for BA.2, and 36 for BA.4/5 in the Dose_0 group (Figure 3(B)). Similar trends of plasma neutralization against variants were also shown in the Dose_1 and Dose_2 groups. Moreover, the titres of nAbs were nearly independent of the initial disease severity and gender of participants in the unvaccinated group and under the same immunization strategy (Figure S4).

To better evaluate the effect of vaccination on the neutralizing antibody response in convalescent individuals at 2-year visit after discharge, we made a direct comparison of these neutralization results by different variants. As shown in Figure 3(C), the GMTs of nAbs for all tested variants (Delta, BA.1, BA.2, and BA.4/5) in 2-dose vaccination convalescents were slightly higher than that in 1-dose vaccination convalescents, and significantly higher than that in unvaccinated convalescents. In some cases, no significant difference was observed among two vaccinated and unvaccinated groups, which may be explained by the decline of antibodies during the long time interval between the sampling and the last vaccination (median: 131 days, IQR: 89-168) (Figure 1, Table S1).

### Decline in plasma neutralization against SARS-CoV-2 variants in COVID-19 convalescents over time after vaccination

**Figure 4.**
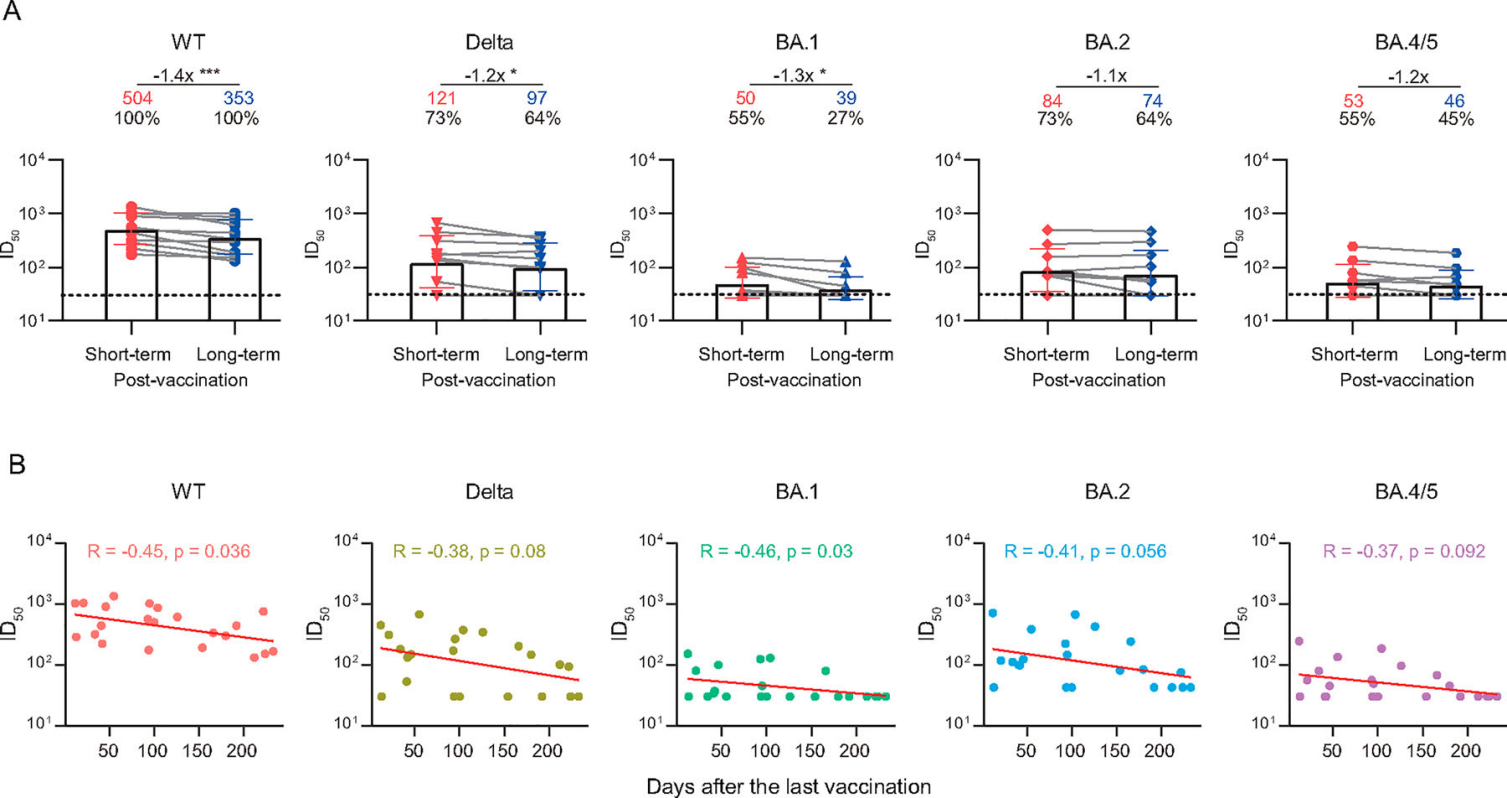
Durability of the broad nAbs post the last vaccination. (A) Paired plasma samples were collected from 11 individuals between the last vaccination and the 2-year follow-up visit, and then tested for neutralizing activities against WT SARS-CoV-2 and variants. The short-term and long-term post-vaccination plasma samples were collected at a median of 42 days (IQR: 28–74) and 180 days (IQR: 140–217) after the last vaccination, respectively. The positive rate, geometric mean, fold-change, and significance of difference were labelled on the top. “−” represents decreased neutralization activity. Statistical significance was determined using two-tailed paired Wilcoxon test. ***P < 0.001; *P < 0.05. The limit of detection was 1:30 dilution. ID_50_ indicates 50% inhibition dilution. (B) Correlation analysis between ID_50_ values against WT SARS-CoV-2 and variants and the days after the last vaccination. Perfect-fit correlation line was included on the plot. The non-parametric Spearman’s correlation coefficients (R) and statistically significant *P* value (p) were provided. The ID_50_ values are means of at least two independent experiments.

Finally, we quantitatively evaluated the degree of antibody decay after vaccination in these convalescent individuals. The plasma samples were additionally collected from 11 individuals at a median of 42 days (IQR: 28–74) after the last vaccination. As shown in Figure 4 and Figure S5, the GMTs of nAbs against WT SARS-CoV-2, Delta, and BA.1 variant were significantly decreased over time, with a median interval of 113 days (IQR: 83–158) from the additional sampling time-point to the 2-year visit. In addition, the correlation analysis also showed the neutralizing activities against WT and BA.1 was negatively correlated to days after the last vaccination.

## Discussion

In this study, we revealed that humoral immune responses to SARS-CoV-2 including circulating antibodies and specific MBCs could be maintained for at least 2 years after discharge, providing the longest follow-up observation in a cohort of COVID-19 convalescents without any vaccination. The kinetics and duration of antibody response in COVID-19 convalescents have always been the focus of attention. Our previous study detected the levels of antibodies over a 6-month follow-up period, and found that anti-RBD IgG antibodies were successfully induced in most subjects, peaked 1 month after symptom onset, and then decreased gradually [7]. Subsequently, Li et al. found that SARS-CoV-2-specific plasma IgG levels were decreased significantly over time within 12 months after infection in 869 COVID-19 convalescents [9]. Later, Yousefi et al. reported the antibodies could be reliably detected in recovered COVID-19 patients for up to 15 months [23]. Furthermore, Choe et al. found that the convalescents with mild COVID-19 could maintain detectable serum IgA and IgG binding antibodies and nAbs against SARS-CoV-2 for 18 months after infection [10]. Taken together, these results above suggested that the durable antibody response could persist for a long time in recovered patients after SARS-CoV-2 infection, which might be comparable to SARS-CoV-1 convalescents for up to 3 years [24]. Therefore, further monitoring for humoral immune memory in these COVID-19 convalescents should be required in the future.

Currently, a greatly increasing concern is the emergence of numerous SARS-CoV-2 variants resulting from the rapid spread and constant evolution of virus. In contrast to the potent neutralization against the WT SARS-CoV-2, varying degrees of immune escape by various variants, such as Beta (B.1.351), Delta (B.1.617.2), and Mu (B.1.621), have been observed in recovered human sera after one year [8, 25]. In particular, with the emergence of various Omicron variants (BA.1, BA.2, and BA.4/5, etc.), the majority of monoclonal nAbs and sera elicited by natural infection and vaccination were escaped [26–29]. It has been reported that less than 50% of the convalescent serum samples could display neutralizing activity against Omicron during 1–12 months after the WT SARS-CoV-2 infection, and their neutralization titres against Omicron were also significantly decreased compared with that against WT, ranging from 11-fold to 32-fold [30–34]. Consistent results were found in this study. Our group evaluated the neutralizing activities of plasma from COVID-19 convalescents at 2 years post discharge against WT, Delta, and Omicron variants (BA.1, BA.2, and BA.4/5). Our findings showed that only 29%-63% of plasma could weakly neutralized Omicron variants, while 75% of which had neutralizing activities against Delta. Omicron variants showed more dramatic reduction of neutralization than Delta variant, which was similar to the previous studies on the sera of COVID-19 convalescents at one year post infection [30, 35].

In addition, several studies demonstrated that plasma neutralizing activity elicited by different clinical vaccines against Omicron variants were severely decreased, and yet vaccine boosters obviously increased the neutralization, including mRNA-1273, BNT162b2, Ad26.COV2.S, and BBIBP-CorV, etc. [31, 36]. In another study, vaccination in prior SARS-CoV-2 infected patients with 2 doses of mRNA vaccines could also increase the neutralizing activity against SARS-CoV-2 variants [37]. Consistent with these previous studies, we found that both positive rate and neutralizing activity of plasma samples maintaining detectable neutralization were increased in the convalescent individuals who received two doses of inactivated vaccines, emphasizing the importance and effectiveness of immunization with SARS-CoV-2 vaccines in these long-term convalescents.

Indeed, it has been well proven that the vaccination is effective in boosting the immune response in recovered COVID-19 patients against circulating SARS-CoV-2 variants [21, 38–40]. After the vaccination with mRNA vaccine, spike-reactive CD4+ and CD8+ T cells and plasma antibody neutralization activities were significantly increased in COVID-19 convalescents [41]. Our previous study also revealed the effectiveness of inactivated vaccine in enhancing the levels of nAbs against various SARS-CoV-2 variants in individuals who have recovered for up to one year [21]. However, the titres of plasma nAbs largely declined over time in both convalescent and vaccinated individuals [42–44]. In this study, our results also showed that plasma neutralization significantly declined after the last vaccination due to the waning immunity. In uninfected populations, the third or even fourth dose of vaccination might be important to prolong the protection and increase the broad-spectrum neutralization against emerging variants [19, 43, 45]. Taken together, this study indicated the need for increased attention to recovered COVID-19 patients and the development of appropriate immunization strategies. A booster vaccination followed by one or two doses may be also needed in these convalescents to enhance and maintain more durable antibody protection against SARS-CoV-2, especially the current circulating Omicron variants.

In conclusion, the humoral immune memory against WT SARS-CoV-2 could remain for up to 2 years in COVID-19 convalescents. However, the plasma neutralization was markedly decreased against various SARS-CoV-2 variants including Delta, BA.1, BA.2, and BA.4/5, suggesting the high risk of reinfection with emerging variants in these convalescents. With the potential to improve plasma neutralization, two or more doses of inactivated vaccines might confer better protection against the currently emerging SARS-CoV-2 variants. Overall, this study provided the longest follow-up visit for monitoring antibody kinetics and neutralizing antibody titres against Omicron variants, which emphasized again the importance of vaccination in individuals who have recovered from the first wave of the COVID-19 pandemic.

## Supplementary Material

Supplemental Material

## Data Availability

We are happy to share reagents and information in this study upon request.
