# Supplementary material for: Omicron variants escape the persistent SARS-CoV-2-specific antibody response in 2-year COVID-19 convalescents regardless of vaccination
Source: Emerg Microbes Infect. 2022 Dec 28;12(1):2151381. doi: 10.1080/22221751.2022.2151381 (PMC9809350; doi:10.1080/22221751.2022.2151381)
Supplement: Supplemental Material [file TEMI_A_2151381_SM7979.zip › Convalescent 2years_Supplementary Material_20221117.docx]

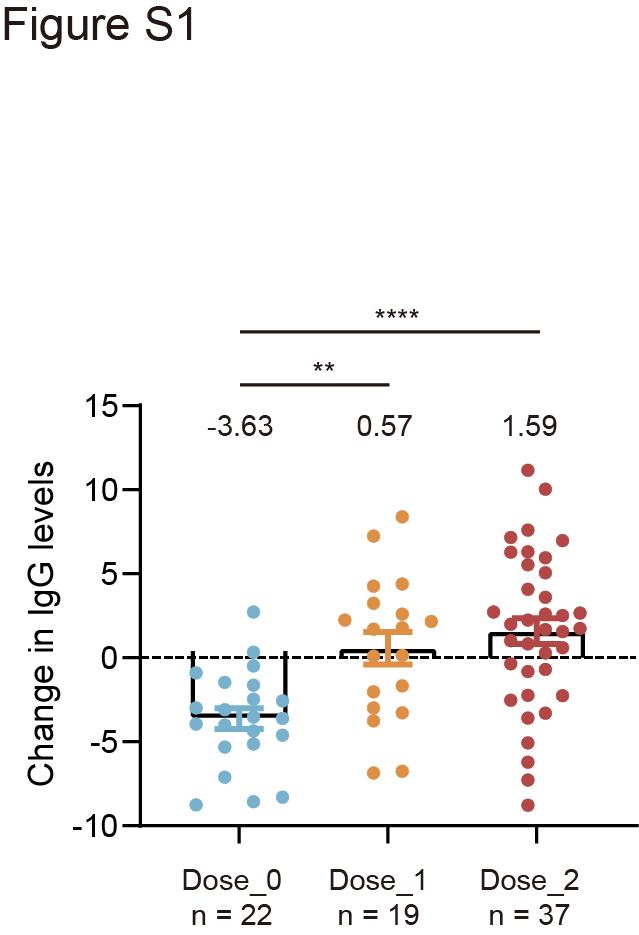


**Figure S1. Changes in anti-RBD IgG levels in the unvaccinated, 1-dose, and 2-dose groups at the 2-year visit relative to the 12-month visit.**

The number of matched samples for each group was labelled at the bottom. The mean value and significance of difference were labelled on the top. Statistical significance was determined using the unpaired Wilcoxon test. **, P < 0.01; ****, P < 0.0001.


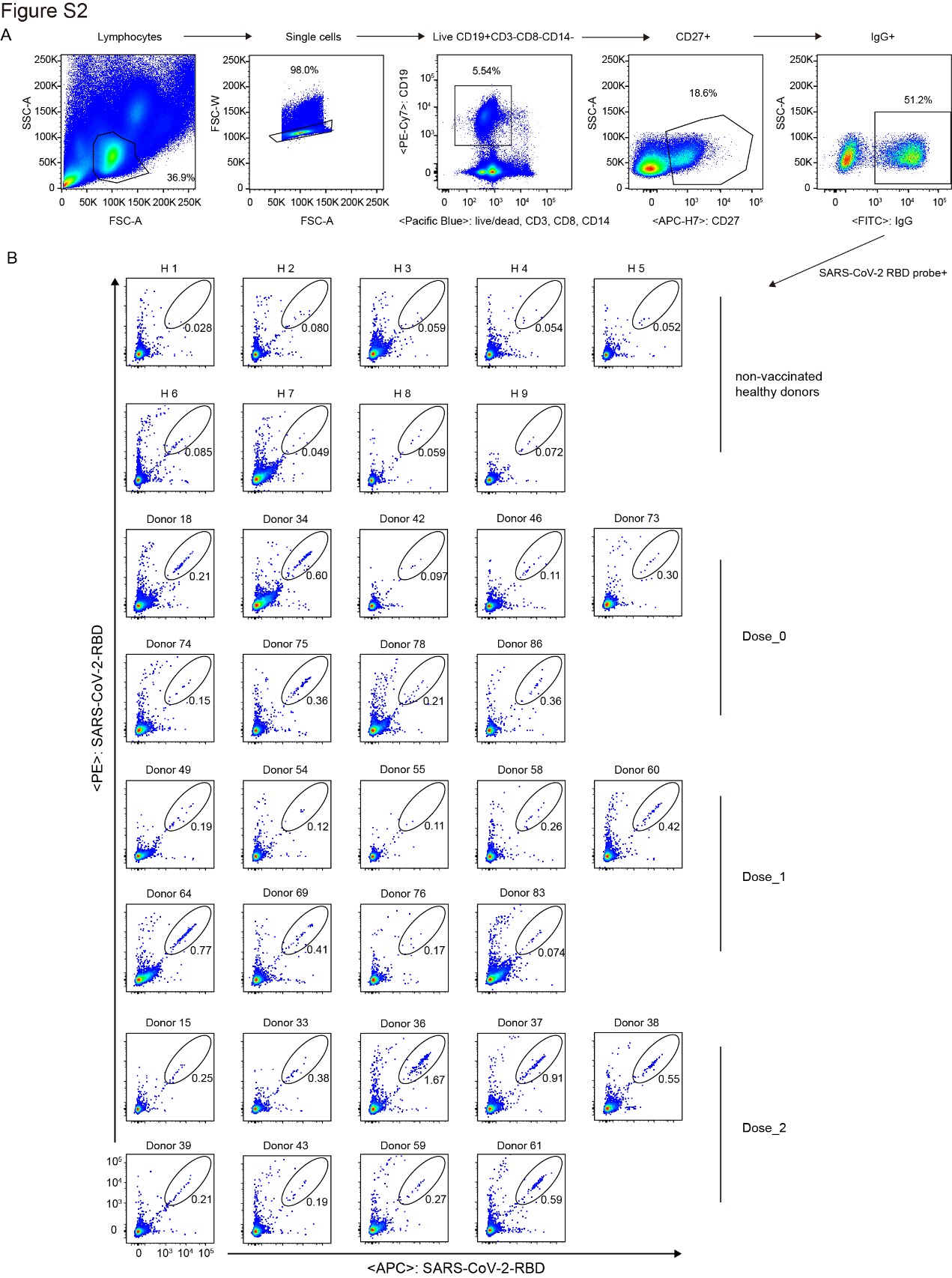


**Figure S2. The gating strategy for identification of SARS-CoV-2 WT RBD-specific memory B cells by FACS.**

**(A)** Single B cells were gated as CD19^+^CD3^-^CD8^-^CD14^-^CD27^+^IgG^+^. **(B)** Flow cytometry showing the percentage of double-positive (APC^+^PE^+^) RBD-binding memory B cells of randomly selected 9 participants in each of non-vaccinated healthy, Dose_0, Dose_1, and Dose_2 groups.
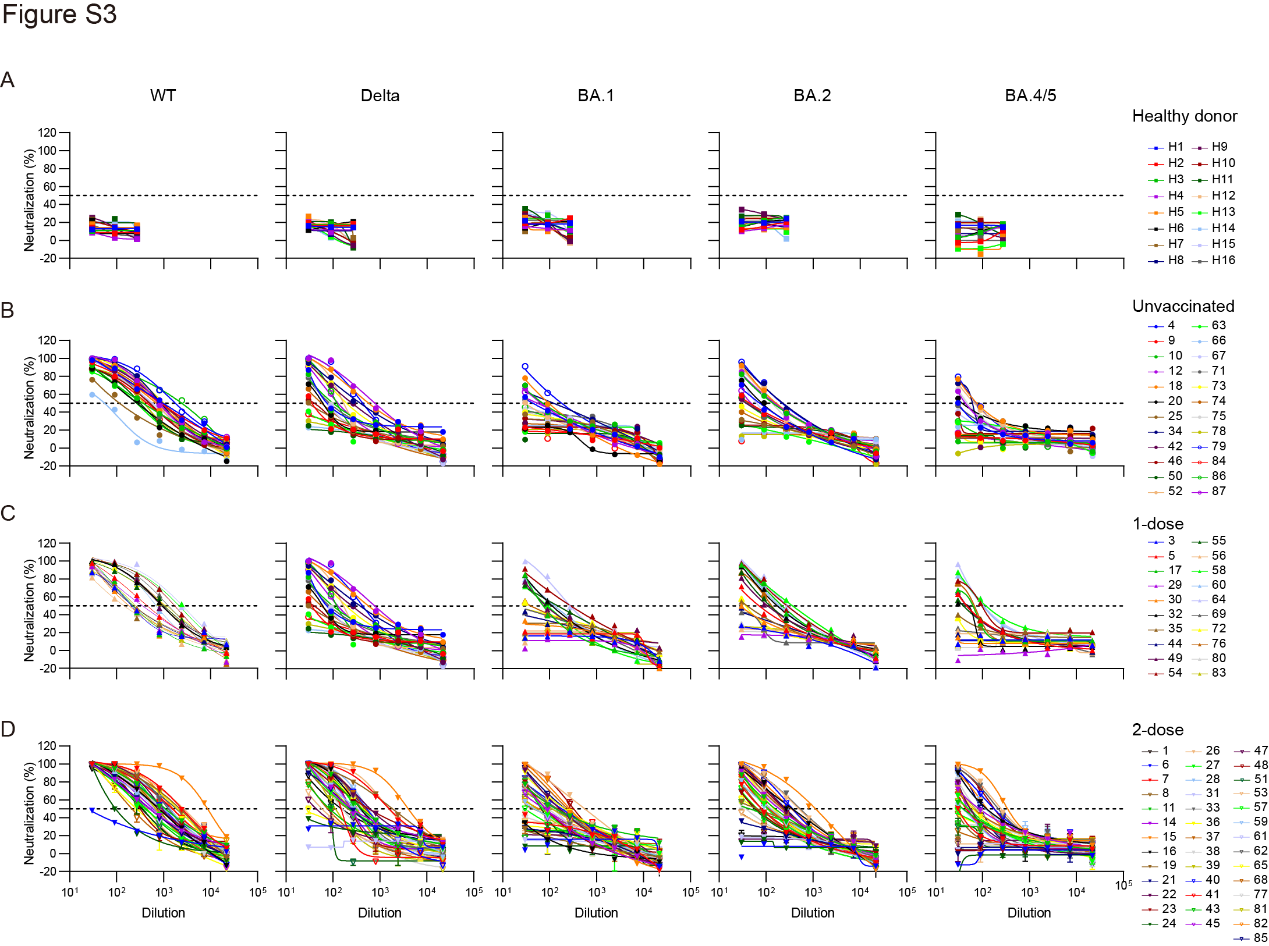


**Figure S3. The plasma neutralization of the (A) non-vaccinated healthy (*n* = 16), (B) unvaccinated (*n* = 24), (C) 1-dose (*n* = 20), and (D) 2-dose individuals (*n* = 40) at the 2-year follow-up time point against the WT SARS-CoV-2 and variants.**

One representative curve from at least two independent experiments was displayed.

**
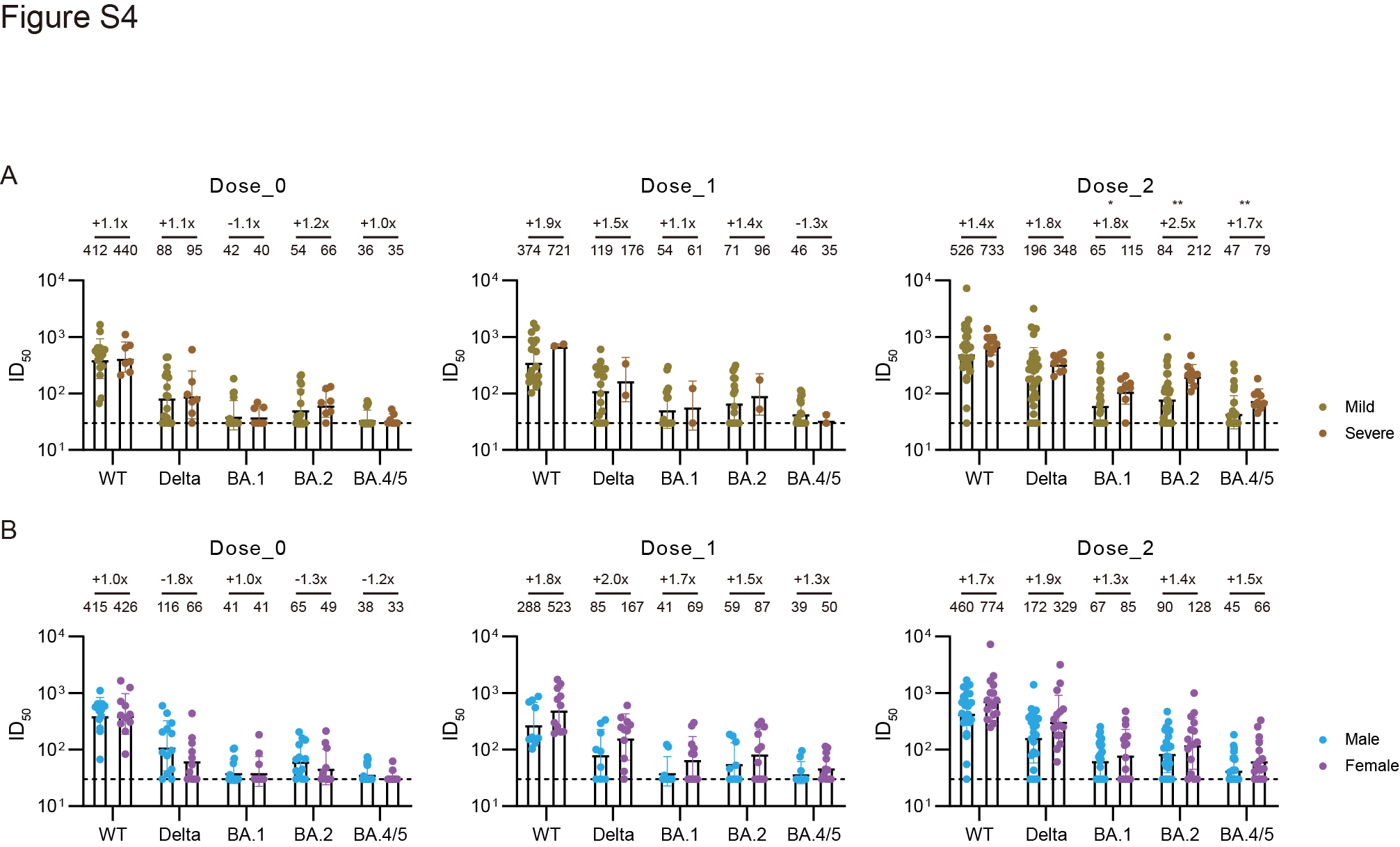
**

**Figure S4. Influences of initial disease severity and gender of the patients on the** **titers of plasma neutralizing antibodies.**

**(A)** Comparison of plasma neutralizing activities against each SARS-CoV-2 strain between mild and severe individuals in each group. **(B)** Comparison of plasma neutralizing activities against each SARS-CoV-2 strain between males and females in each group. The geometric mean, fold-change, and significance of difference were labelled on the top. “-” represents decreased neutralization activity, and “+” represents increased neutralization activity. The unpaired Wilcoxon test was performed. *, P < 0.05; **, P < 0.01. The limit of detection was 1:30 dilution. ID_50_ indicates 50% inhibition dilution.

**
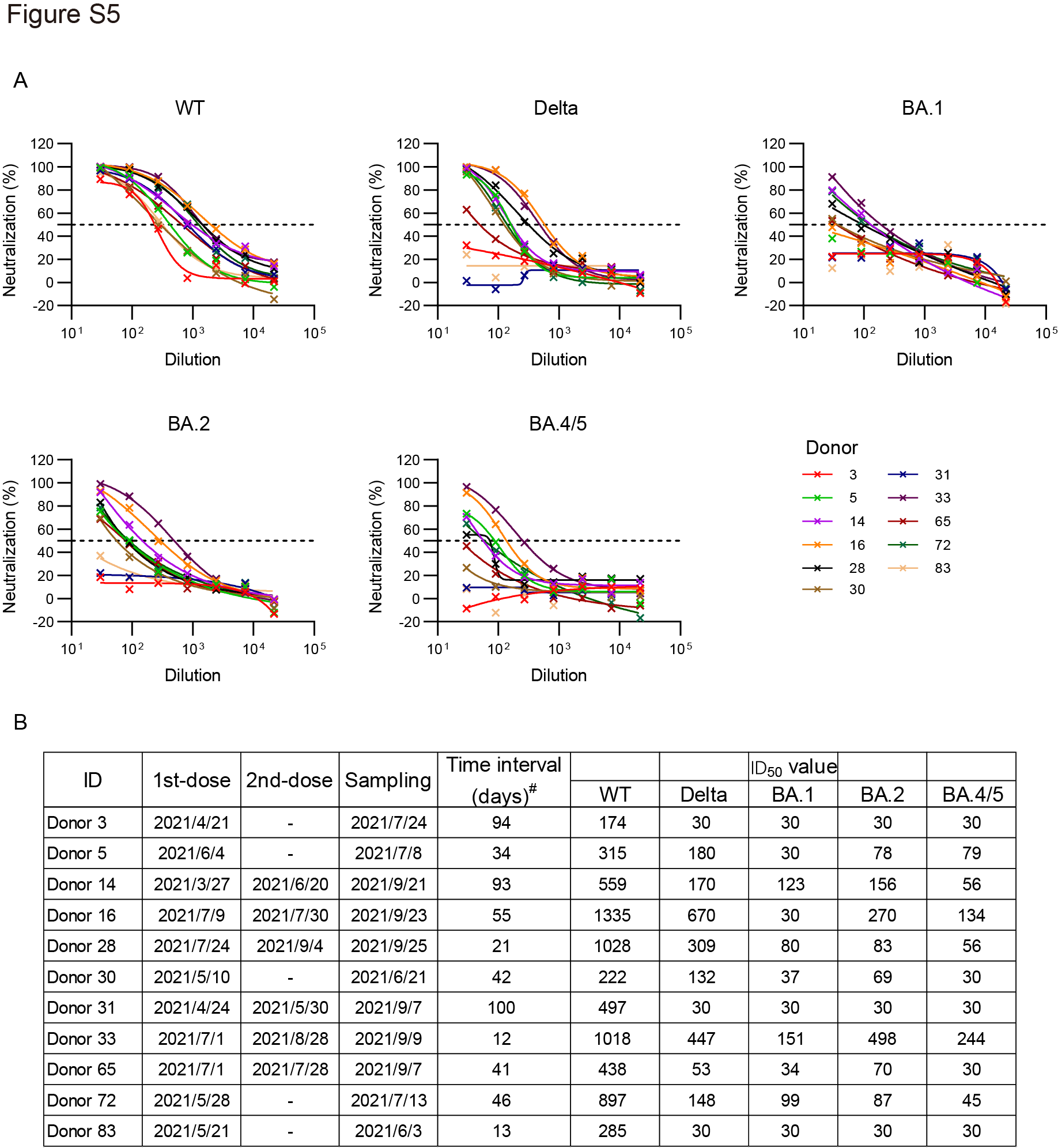
**

**Figure S5. The plasma neutralization of 11 individuals post vaccination against the WT SARS-CoV-2 and variants.**

**(A)** Plasma samples of 11 individuals were collected after the last vaccination. One representative curve from at least two independent experiments was displayed. **(B)** ID_50_ values of each plasma against each SARS-CoV-2 strain. The data was shown in mean of at least two independent experiments. The data below the limit of detection (1:30) was set to 30 for visualization. #: the time interval between sampling and the last immunization. -: not available.

**Table S1. Demographic characteristics, time points of vaccination, detailed vaccine information, and blood sampling of enrolled participants in this study.**


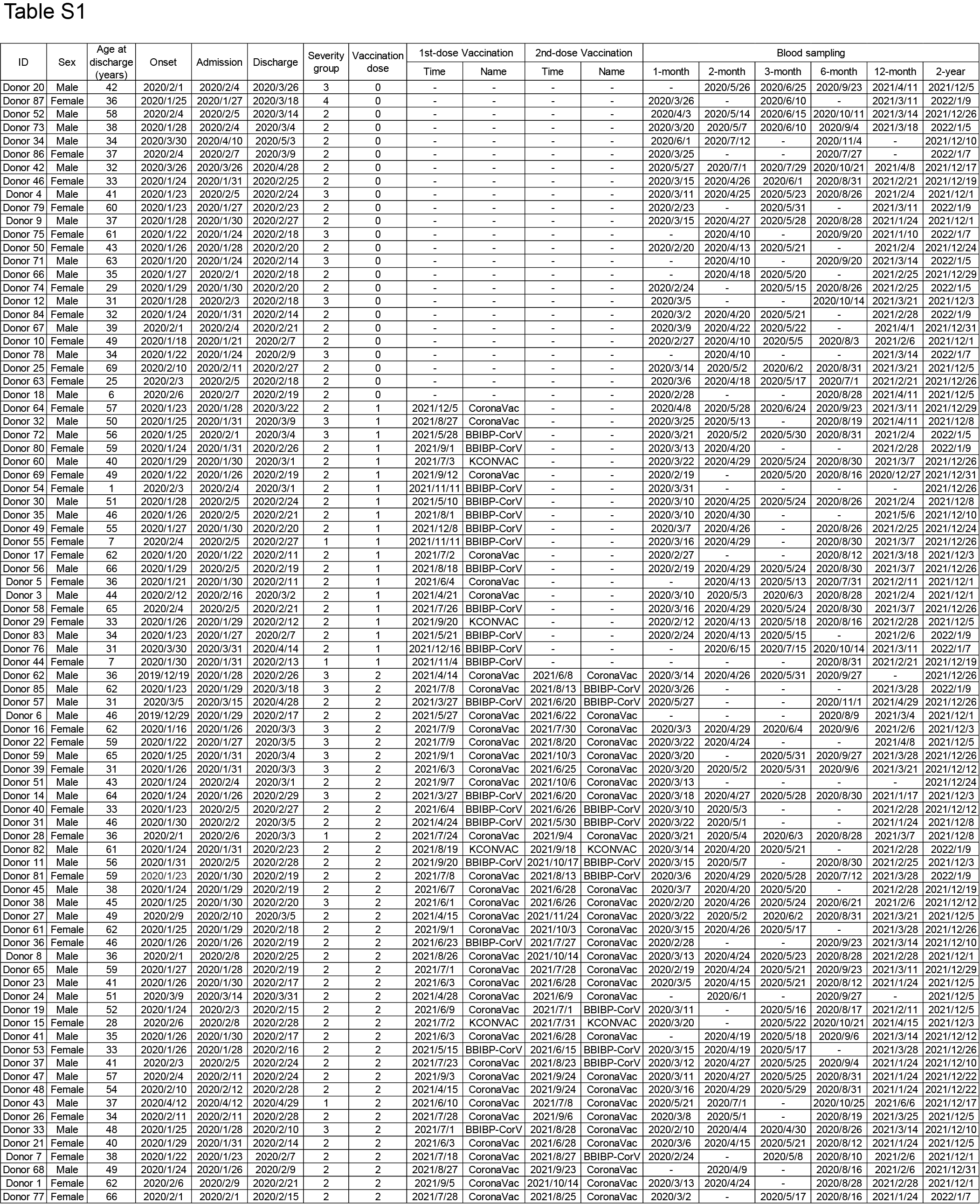


-: not available.

**Table S2. Anti-RBD IgG levels in plasma sample of individuals at six follow-up time points.**


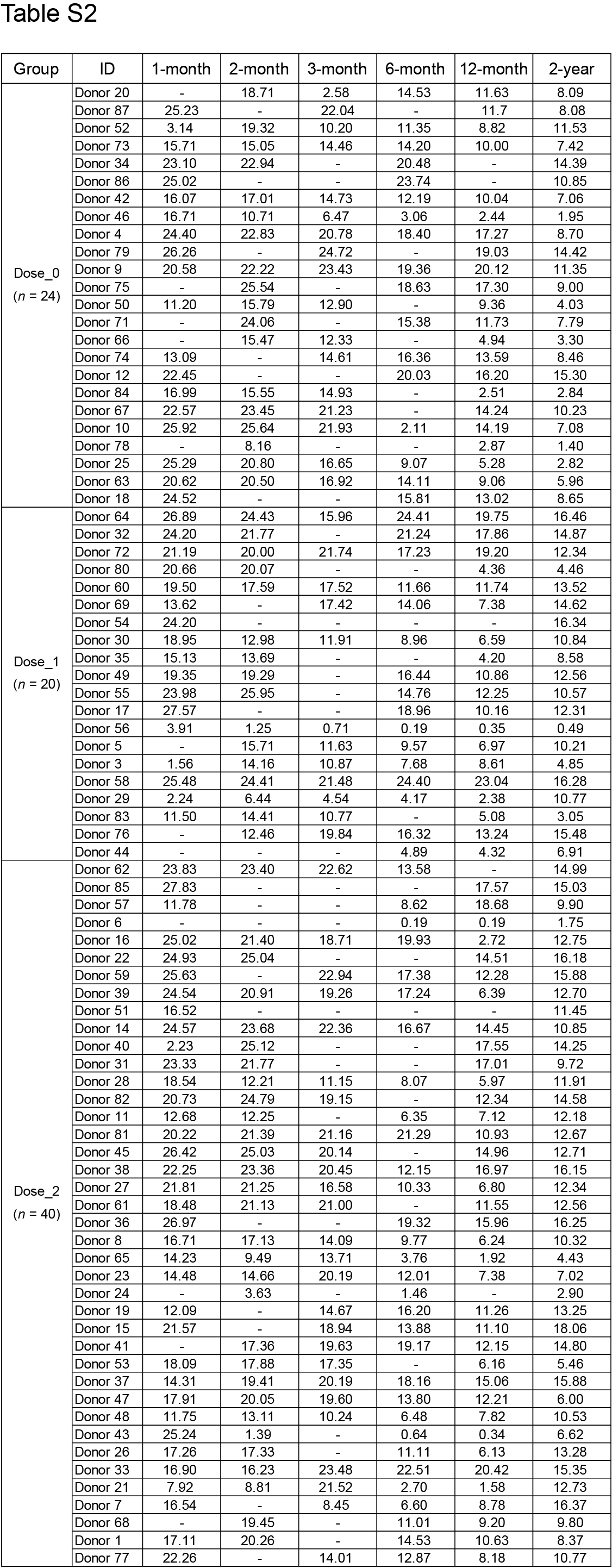


-: not available.

**Table S3.** **The ID_50_ values of each plasma from 84 individuals at the 2-year follow-up time point against WT SARS-CoV-2 and variants.**


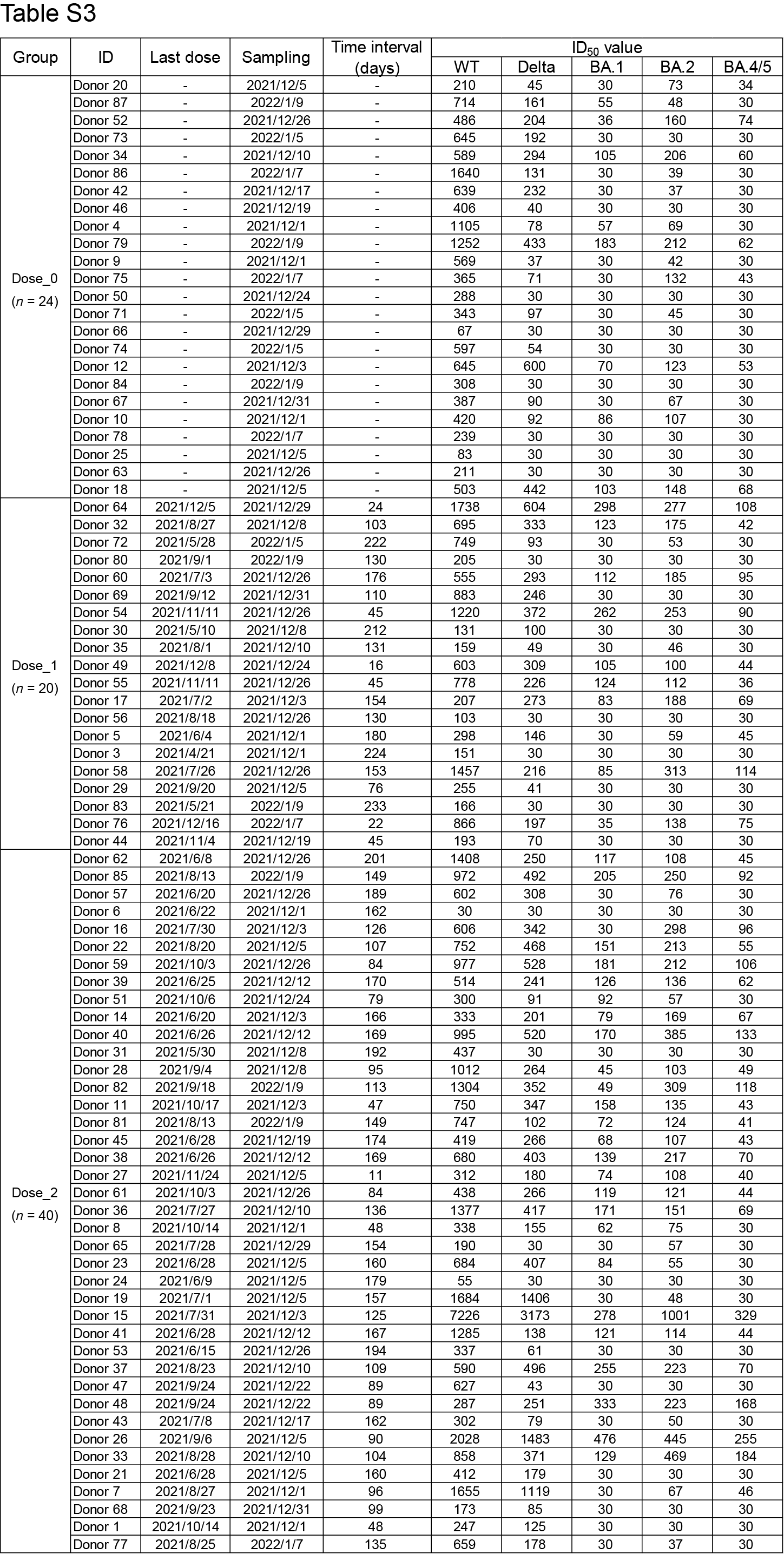


The data was shown in mean of at least two independent experiments. The data below the limit of detection (1:30) was set to 30 for visualization. -: not available.
